# Risk of type 2 diabetes in metabolically healthy people in different categories of body mass index: an updated network meta-analysis of prospective cohort studies

**DOI:** 10.15171/jcvtr.2019.43

**Published:** 2019-10-24

**Authors:** Somayeh Tajik, Atieh Mirzababaei, Ehsan Ghaedi, Hamed Kord-Varkaneh, Khadijeh Mirzaei

**Affiliations:** ^1^Department of Community Nutrition, School of Nutritional Sciences and Dietetics, Tehran University of Medical Sciences (TUMS), Tehran, Iran; ^2^Student’s Scientific Research Center, Tehran, Iran; ^3^Department of Cellular and Molecular Nutrition, School of Nutritional Sciences and Dietetics, Tehran University of Medical Sciences, Tehran, Iran; ^4^Student Research Committee, Department of Clinical Nutrition and Dietetics, Faculty of Nutrition and Food Technology, Shahid Beheshti University of Medical Sciences, Tehran, Iran

**Keywords:** BMI, Obesity, Metabolic Healthy, Metabolic Unhealthy, Metabolic Syndrome, Diabetes Mellitus Type 2, T2DM

## Abstract

***Introduction:*** Risk of diabetes mellitus type 2 (T2DM) is variable between individuals due to different metabolic phenotypes. In present network meta-analysis, we aimed to evaluate the risk of T2DM related with current definitions of metabolic health in different body mass index (BMI) categories.

***Methods:*** Relevant articles were collected by systematically searching PubMed and Scopus databases up to 20 March 2018 and for analyses we used a random-effects model. Nineteen prospective cohort studies were included in the analyses and metabolically healthy normal weight (MHNW) was considered as the reference group in direct comparison for calculating indirect comparisons in difference type of BMI categories.

***Results:*** Total of 199403 participants and 10388 cases from 19 cohort studies, were included in our network meta-analysis. Metabolically unhealthy obesity (MUHO) group poses highest risk for T2DM development with 10 times higher risk when is compared with MHNW (10.46 95% CI; 8.30, 13.18) and after that Metabolically unhealthy overweight (MUOW) individuals were at highest risk of T2DM with 7 times higher risk comparing with MHNW (7.25, 95% CI; 5.49, 9.57). Metabolically healthy overweight and obese (MHOW/MHO) individuals have (1.77, 95% CI; 1.33, 2.35) and (3.00, 95% CI; 2.33, 3.85) risk ratio for T2DM development in comparison with MHNW respectively.

***Conclusion:*** In conclusion we found that being classified as overweight and obese increased the risk of T2DM in comparison with normal weight. In addition, metabolically unhealthy (MUH) individuals are at higher risk of T2DM in all categories of BMI compared with metabolically healthy individuals.

## Introduction

The prevalence of diabetes has been increasing, and it is been rising among overweight and obese people.^1^ Obesity is a risk factor for developing diabetes, postulated by mediation with pathways related with fat tissue metabolism include abnormal beta cell dysfunction and aggravated insulin resistance (IR).^2^ Metabolically healthy obese (MHO) is the one of subcategories of obesity without lipid disorders, IR or hypertension.^3^ Generally, there is no evidence that these individuals are permanently protected from the risk of obesity-related co-morbidities.^4^

Several prospective cohort studies have shown the combined effect of a higher body mass index (BMI) and metabolic abnormalities in the development of diabetes. Some of these have reported that MHO individuals are not at increased risk for diabetes mellitus type 2 (T2DM) in comparison with metabolically healthy non-obese subjects^5-7^ and might not benefit from lifestyle interventions. In return, some articles have provided evidences that MHO phenotype might be associated with a significant increased risk of the development of diabetes in comparison with metabolically healthy non-obese subjects.^8-12^ This controversy in these studies may originate from diverse of ethnicity in different populations.^13^ and effect of ethnicity on lean body mass.

These data suggested that further studies are required to evaluate the risk of diabetes according to the phenotypes of obesity. Two meta-analyses up to 2014^14,15^ suggested that MHO phenotype might be associated with a significant increased risk of diabetes development in comparison with metabolically healthy non-obese subjects, while after these meta-analyses several studies have been published.^7,12,16,17^

Therefore, this network meta-analysis updated studies up to March 20, 2018 based on ethnicity in different population, and aimed to provide more clear evidence on the risk of diabetes development based on phenotypes of obesity.

## Methods

This network meta-analysis according to the meta-analysis of observational studies in epidemiology (MOOSE) guidelines.^18^

### Search strategy

All relevant published cohort studies that tested the associations of different phenotypes of BMI with risk of T2DM were searched. These studies systematically searched throughout PubMed and Scopus databases up to March 20, 2018 and it was conducted by using MESH and non-MESH keywords and did not use date and language restrictions. The terms were used in the electronic search included “Adiposity”[Mesh]) OR “Adiposity”[Title/Abstract] OR [“Obesity”[Mesh] OR “Obesity”[Title/Abstract] OR “Body Mass Index”[Mesh] OR “Body Mass Index”[Title/Abstract]) OR “BMI” OR “Overweight”[Mesh] OR “Overweight”[Title/Abstract] OR (“Obesity, Metabolically Benign”[Mesh]) OR “benign obesity”[Title/Abstract] OR “healthy obesity”[Title/Abstract]) OR “metabolically healthy obesity”[Title/Abstract]) OR “metabolically benign obesity”[Title/Abstract]) OR (“obesity”[Title/Abstract]) AND “metabolically healthy”[Title/Abstract]) OR (“obesity”[Title/Abstract]) AND “metabolically benign”[Title/Abstract]] AND (“Diabetes Mellitus”[Mesh]) OR “Diabetes Mellitus”[Title/Abstract]) OR “Diabetes Mellitus, Type 2”[Mesh]) OR “Diabetes Mellitus Type 2”[Title/Abstract]) OR “Diabetes Mellitus Type II”[Title/Abstract]) OR “T2DM”[Title/Abstract]) OR “T2D”[Title/Abstract]) OR “type 2 diabetes”[Title/Abstract]) OR “type 2 diabetes mellitus”[Title/Abstract]) OR “Type II diabetes mellitus”[Title/Abstract]) OR “Type II diabetes”[Title/Abstract]) OR Diabet*[Title/Abstract]) OR “Noninsulin-dependent diabetes mellitus”[Title/Abstract]. In addition, the reference list of obtained articles was manually reviewed.

### Eligible criteria

Articles were considered as qualified for this network meta-analysis if they met the following criteria: had prospective cohort design; tested association of metabolically healthy (MH) or metabolically unhealthy (MUH) phenotypes of BMI with risk of T2DM; reported hazard ratios (HRs), odds ratios (ORs) or relative risks (RRs) for risk of T2DM; stratified subjects according to weight categories; describes criteria used for defining individuals as MH or MUH and included healthy subjects over 18 years. Studies were excluded if they have following criteria; case reports, cross sectional, literature reviews, animal studies, republished data and grey literature including; congress abstracts, dissertations, and patents. We did not find grey literature in references and related articles. The information could not be extracted were; non-English studies and those which did not report risk of T2DM.

### Study selection

Titles and abstracts of all collected papers were reviewed separately by 2 reviewers (AM and KH-M). Studies that did not meet the eligibility criteria were excluded by using a screening form with a hierarchical approach based on population, study design, or exposure and outcome. Then, full articles of eligible studies were entered to a second evaluation by the same reviewers. Both of reviewers agreed this screening.

### Data extraction

Two reviewers (AM and KH-M) separately extracted data from the included publications. The information acquired from each study includes: authors’ name, publication year, country of participants, ethnicity, mean range of age, study sample size, criterion used to define weight of participants, number of participants/ cases in each category, follow-up duration, definition of metabolic phenotypes healthy /unhealthy, confounding factors that were adjusted for in the multivariable analysis, and the HRs/RRs/ORs with corresponding 95% confidence intervals (CI).

### Quality assessment for individual studies

Two assessors (AM and Kh-M) valued the quality of each selected study using the Newcastle-Ottawa scale (NOS) for cohort studies.^19^ Studies with a score of 3 to 7 as moderate quality and those with a score of seven or higher were reflected as high quality.

### Statistical analysis

The present network meta-analysis was performed by using pairwise and network commends in Stata (version 14).^20,21^ A random-effects model was conducted to calculate direct and indirect comparisons. The metabolically healthy normal weight (MHNW) was considered as the reference group in direct comparison for obtained indirect comparisons in difference type of BMI categories. Pooled (RRs and 95% CI were calculated using random-effects. The if plot command was employed to produce the inconsistency plot assuming loop-specific heterogeneity estimate and demonstration the exp (IF).^22^ Differences in effect estimates between direct and indirect comparisons (statistical inconsistency) in the data was evaluate with local and global methods.^23^ Moreover, we conducted subgroup analysis based on different population to better clarify the risk of diabetes incidence on BMI categories.

## Results

### Study screening

**Figure. 1:**
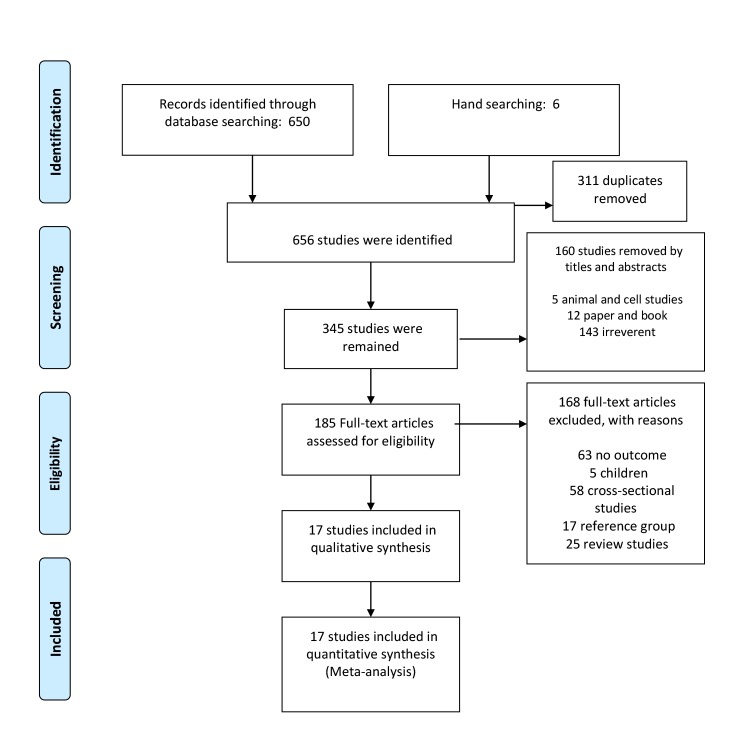
PRISMA flowchart of the study.

The flowchart of article selection is shown in Figure 1, 650 references were identified through electronic searches and 6 by manual searches, of which 311 were duplicates and 160 were non-relevant, which were excluded on the initial screening of titles and abstracts. Of the remaining, another 166 articles were excluded (detailed reasons for the exclusions are given in Figure 1). Finally, 19 studies involving a total of 199 403 participants and 10 388 cases were included in our meta-analysis (see Supplementary file 1, Table S1).

### Study characteristics

Nineteen studies reported the risk of T2DM diseases in 199403 participants and 10388 cases. These studies were published between May 30 2006 and 25 June 2017. The age of individuals was 18 to 50 years old. Among the included studies, eleven were from the Asian populations^10,12,16,17,24-30^ two from American populations,^5,9^ three from European populations^8,11,31^ and one, was from Australia populations. ^6^ Subjects were then classified according to their BMI as normal weight, overweight and obese, only in study of Luo et al,^17^ body fat percentage (BF %) >25 % for men or BF (%) >35% for women were defined as being obese. The duration of follow-up differs from 2 to 20 years. Three studies were conducted on male.^8,16,28^ The results in the most of the studies were adjusted for potential confounders, including sex, age, smoking status and physical activity. All studies were cohort. Furthermore, the reference category was a MHNW phenotype in all studies. Table S1 summarizes the characteristics of the included studies. The definition of MH/MUH phenotypes was based on criteria by the Adult Treatment Panel III (ATPIII),^5,9,12,28,30,31^ International Diabetes Federation (IDF),^6,26,27^ joint committee for developing Chinese guidelines (JCDCG),^17^ components metabolic syndrome (MetS),^11^ modify ATPIII,^8^ Harmonized metabolic syndrome,^10,24^ modify (American Heart Association) AHA,^25^ Wildman^16,29^ and Homeostatic model assessment –insulin resistance (HOMA-IR).^9^ In quality assessment of articles two studies received 10 score ^27,31^ and all other studies received 8 to 9 score.^5,6,8,12,16,17,24-26,28-30^

### BMI and risk of T2DM

**Figure. 2:**
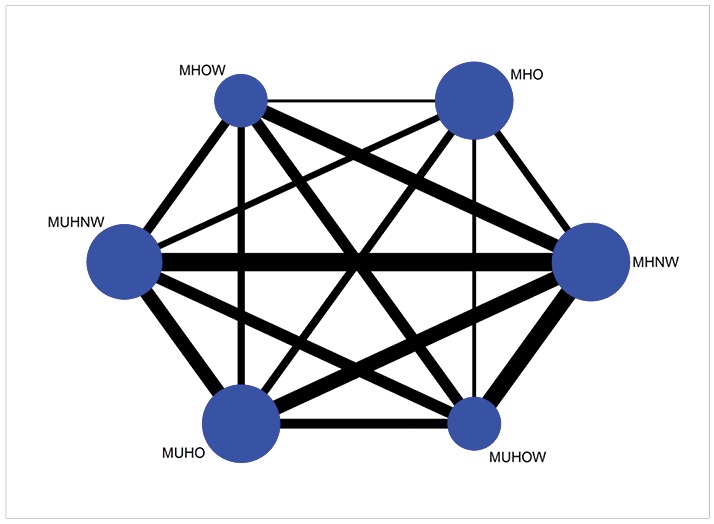
Network of comparisons included in the analyses. MHNW, metabolically healthy normal weight; MHO, metabolically healthy obesity; MUHO, metabolically unhealthy obesity; MHOW, metabolically healthy overweight; MUHOW, metabolically unhealthy overweight’ MUHNW, metabolically unhealthy normal weight.

**Figure. 3:**
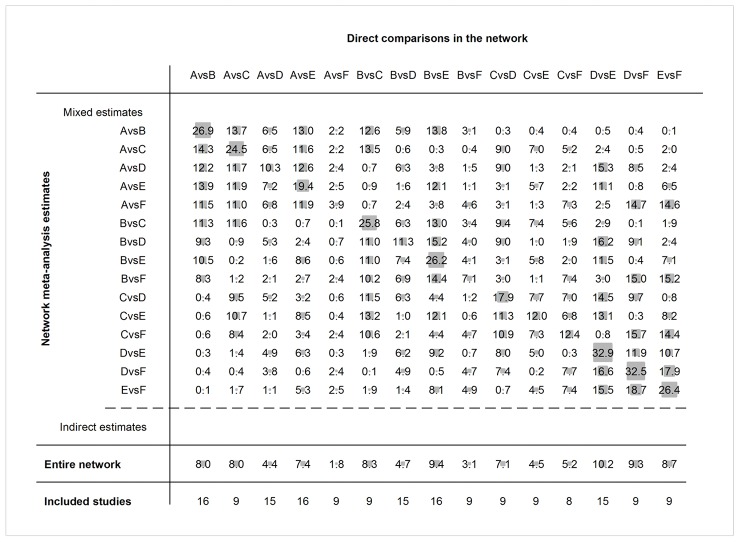
Contribute figure. Note: A: metabolically healthy normal weight, B: metabolically healthy obesity, C: metabolically healthy overweight, D: metabolically unhealthy normal weight, E: metabolically unhealthy obesity, F: metabolically unhealthy overweight.

Figure 2 shows the network diagrams of direct comparison for different BMI status, with the number of studies reflected by the size of the ages, and the number of patients reflected by the size of the nodes. Six comparisons among BMI groups were considered in our NMA; MHNW was the most “common comparator”. The highest number of comparisons included MHNW compared with MHO (n = 16), MHO compared with metabolically unhealthy obesity (MUHO) (n = 16), MHNW weight compared with metabolically unhealthy normal weight (MUHNW) (n = 15), MHO compared with MUHNW (n = 15), and MUHNW compared with metabolically unhealthy obesity (MUHO) (n = 15), for observe more information see contribute Figure 3.

**Figure. 4:**
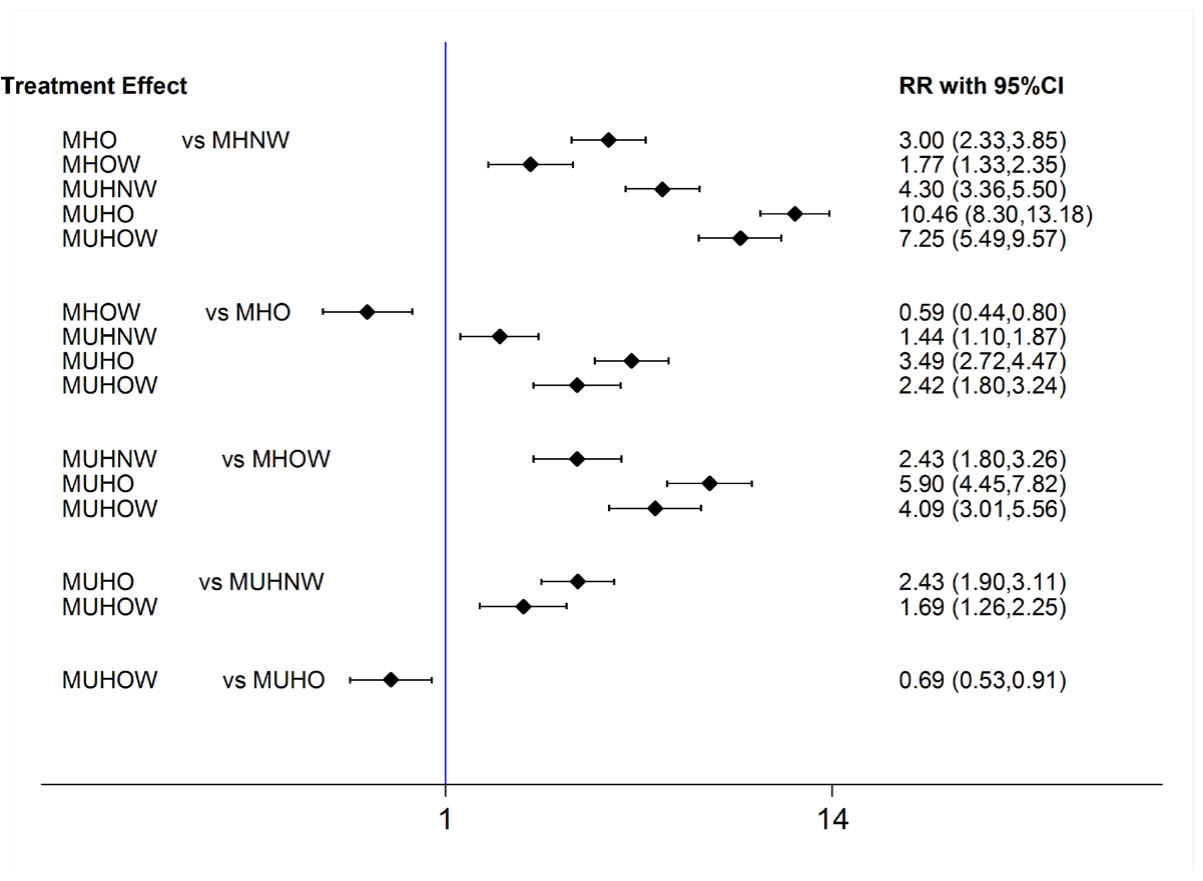
Interval plot. MHNW, metabolically healthy normal weight; MHO, metabolically healthy obesity; MUHO, metabolically unhealthy obesity; MHOW, metabolically healthy overweight; MUHOW, metabolically unhealthy overweight; MUHNW, metabolically unhealthy normal weight.

Nineteen articles were included with 158,583 participants and 7675 diabetes events. The pooled estimates of NMA combining direct and indirect evidence the BMI on risk of T2DM are presented in Table 1 and Figure 4 (Interval plot).The overall results of risk of T2DM were significantly increased in MUHO (RR: 10.46; 95% CI: (8.30-13.18), metabolically unhealthy overweight (MUHOW) (RR: 7.25; 95% CI: (5.49-9.57), MUHNW (RR: 4.30; 95% CI: (3.36-5.50), MHO (RR: 3.00; 95% CI: 2.33-3.85), metabolically healthy overweight (MHOW) (RR: 1.77; 95% CI: 1.33-2.35) compared with MHNW.

**Table 1:** Network estimated relative risk (95% CI) of BMI categories on incidence of diabetes

**_MUHOW_**	**_MUHO_**	**_MUHNW_**	**_MHOW_**	**_MHO_**	**_MHNW_**
MUHOW	1.44 (1.10,1.90)	0.59 (0.44,0.79)	0.24 (0.18,0.33)	0.41 (0.31,0.55)	0.14 (0.10,0.18)
0.69 (0.53,0.91)	MUHO	0.41 (0.32,0.53)	0.17 (0.13,0.22)	0.29 (0.22,0.37)	0.10 (0.08,0.12)
1.69 (1.26,2.25)	2.43 (1.90,3.11)	MUHNW	0.41 (0.31,0.55)	0.70 (0.54,0.91)	0.23 (0.18,0.30)
4.09 (3.01,5.56)	5.90 (4.45,7.82)	2.43 (1.80,3.26)	MHOW	1.69 (1.25,2.28)	0.56 (0.42,0.75)
2.42 (1.80,3.24)	3.49 (2.72,4.47)	1.44 (1.10,1.87)	0.59 (0.44,0.80)	MHO	0.33 (0.26,0.43)
7.25 (5.49,9.57)	10.46 (8.30,13.18)	4.30 (3.36,5.50)	1.77 (1.33,2.35)	3.00 (2.33,3.85)	MHNW

MHNW, metabolically healthy normal weight; MHO,metabolically healthy obesity; MUHO,metabolically unhealthy obesity; MHOW,metabolically healthy overweight; MUHOW,metabolically unhealthy overweight; MUHNW,metabolically unhealthy normal weight

### Subgroups analysis based on ethnicity

In the subgroups analysis the overall results of risk of T2DM were stronger based on ethnicity, in whichrisk of T2DM was stronger in MUHO than MUHOW, MUHNW, MHO, MHOW and MHNW, respectably (Tables S2- S4).

### Network inconsistency

Two approaches were conducted to assess the inconsistency in the NMA. By using a loop-specific approach, each IF (ratio of odds ratios) had a 95% CI, indicating little evidence of loop inconsistency between direct and indirect evidence (Figure S1). The node-splitting approach indicated that no significant inconsistency was observed within the networks (Table S5).

### Publication bias

**Figure. 5:**
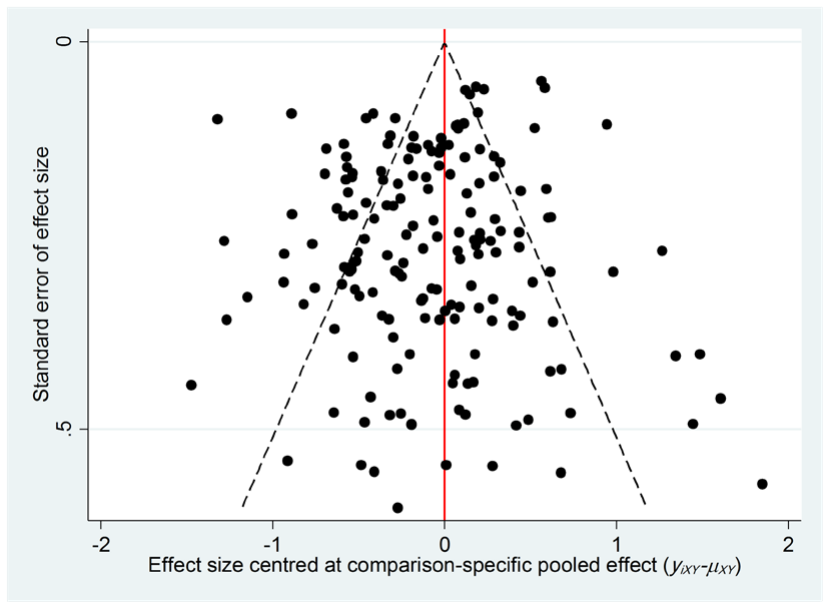
Comparison-adjusted funnel plot of publication bias in relations of diabetes incidence in BMI categories.

Publication bias assessment with funnel plot for the primary outcomes of risk of diabetes in BMI categories did not indicate publication bias in the meta-analysis and was roughly symmetrical (Figure 5).

## Discussion

Here in present network meta-analysis we aimed to evaluate the risk of T2DM related with current definitions of metabolic health in different BMI categories. The main finding of present study is that being classified as overweight and obese in categories of BMI increased the risk of T2DM in comparison with normal weight. In addition MUH individuals are at higher risk of T2DM in all categories of BMI compared with MH individuals. In fact all groups within BMI and MH categories had higher risk when compared with MHNW group. MUHO group demonstrates the highest risk for T2DM development when compared with MHNW with 10 times higher (10.46 95% CI; 8.30, 13.18) and after that MUOW individuals were at highest risk of T2DM with 7 times higher risk when compared with MHNW .Metabolically healthy overweight and obese individuals has (1.77, 95% CI; 1.33, 2.35) and (3.00, 95% CI; 2.33, 3.85) risk ratio for T2DM development in comparison with MHNW respectively. Also MUHNW individuals has higher risk (4.30, 95% CI; 3.36, 5.50) in comparison with MHNW subjects. The 4 times higher risk of MUHNW compared with MHNW subjects (4.30, 95% CI; 3.36, 5.50) was also higher than MHOW and MHO risk of T2DM development in comparison with MHNW individuals. Eventually MUHO (3.49, 95% CI; 2.72,4.47) and MUHOW individuals (4.09, 95% CI; 3.01,5.56) although has higher risk for T2DM development compared with same BMI categories including MHO and MHOW respectively, but the risk was definitely lower than what is obtained for MUHNW compared with MHNW individuals which must be considered in future studies.

Although MHO group had 3 times higher risk of T2DM compared with MHNW as reported previously, their risk also were lower significantly with 10 times higher risk in MUO group. Interestingly these findings highlight the effect of metabolic health status of individuals for predicting T2DM development in populations; metabolic abnormalities may be more contribute to the development of T2DM rather than exclusive anthropometric measurement like BMI. Here we showed increased risk of T2DM development in MHO individuals which is consistent with some,^32-37^ but not all previous studies.^6,38-41^ In fact BMI could be a risk factor for T2DM regardless of the metabolic status of the participants. In a meta-analysis MHO individuals had an increased risk of develop diabetes compared with MHNW individuals.^42^ However both the Framingham ^38^ and an Australian cohort study ^6^ reported that MHO and MHNW individuals had similar risk of developing diabetes during 11-year period.

Obesity might not be diabetogenic per se, and not all MH individuals with obesity are at the same risk of diabetes onset. Adipose tissue as an endocrine organ produces different adipocytokines like leptin and adiponectin, which could be causally associated with wide range of metabolic diseases.^43^ On the other hand, abundance adipose tissue can increase inflammatory status and oxidative stress by secreting markers like CRP, interleukin-6, monocyte chemotactic protein-1 (MCP-1), and tumor necrosis factor-α (TNF-α), all of which have been suggested to be contributed in the pathogenesis of diabetes.^44^ In addition due to excess of adipose tissue accumulation pancreatic fat, may contribute to β-cell dysfunction and probably to the following development of T2DM.^45,46^ However, it has been suggested that the MHO phenotype is transient and ultimately progresses to overt metabolic abnormalities in significant percentage of individuals over time.^47^ Thus additional mechanistic studies are required to clarify the relationship between the MHO phenotype and the impact of changes in obese phenotype overtime and the increased risk of diabetes.

We showed that MUHNW individuals have higher risk for T2DM development. Actually MUHNW subjects represent another spectrum of obesity.^48^ These individuals show more adiposity, more abdominal fat distribution, and dyslipidemia than MHNW individuals.^49^ It has been proposed that disturbance in fat storage in adipose tissue could elucidate the increase in triglyceride levels and the ectopic fat deposition in the liver and muscle.^50^ Furthermore MUHNW individuals have decreased compensatory insulin response in comparison with MHNW individuals.^51^ Previous studies indicate that MUHNW individuals are at increased risk of T2DM, cardiovascular diseases and mortality ^52-54^ which is aligned with our data in terms of the risk of developing T2DM.

The definitions of overweight and obesity are according to information from European populations.^55^ Ethnic differences in the prevalence of T2DM reported in several countries. Higher T2DM prevalence in Asia and Africa and their descendants reported in comparison with whites.^56^ Asian individuals are more expected to have a higher percentage of fat or visceral adipose tissue at a given BMI than Europeans.^57^ So that the associations of BMI and health outcomes may differ between Asian and European populations.^58^ Asians have shown to have an increased risk of T2DM in a comparatively low BMI.^58^ Our results indicated that the risk for developing T2DM in increased BMI categories defined by lower cutoff points for Asians were to some extent higher than those obtained using BMI criteria for European populations but lower than American population. However, in metabolically unhealthy groups these ratios changed totally with a considerable higher risk in American population (18 times higher risk (18.76, 95% CI: 12.62, 27.89) but approximately same risk between European and Asian population (about 10 times higher). The ethnic dissimilarity may associate to the genetic factors, lifestyle factors or body composition.

Obesity reported with different incidence all over the world from Asia to America and Europe which can affect T2DM development. However, several studies showed significant differences regarding insulin resistance and hyperinsulinemia independent of obesity in different ethnics like non-Hispanic, Asian American and native Americans,^59^ Differences in brown adipose tissue in South Asians may contributed to an reduced energy expenditure and T2DM.^56^ BF (%) in Indian individuals is higher than that in European at a given BMI; this ‘thin-fat’ phenotype is associated with higher insulin resistance. Recently Indian people with T2DM reported to have lower beta cell function compared with Europeans which is originates during intrauterine development, and not only diet; this fact support the ‘thrifty phenotype’ hypothesis.^60^ Diet may also play a fundamental role in intergenerational vulnerability to T2DM. Maternal vitamin B12 deficiency and hyperhomocysteinemia are related to IR and adiposity in childhood.^61^ Macronutrient imbalances and higher glycemic index affect IR.^62^ Different sphingolipid, plasma amino acid especially branched-chain amino acids, and mono- and polyunsaturated acylcarnitine and plasma levels reported in different races. Differences in these metabolites were associated with cumulative and incident T2DM.^56^ Genetic background also has been proposed as a possible description for ethnic differences in DM risk; as more than 40 loci are associated with an increased risk for T2DM regardless of race or ethnicity and due to few large-scale genetic studies future finding for genome wide association studies of T2DM is needed. Different HbA1c level also reported in different possibly due to erythrocyte differences.^59^ All these factors affect the possible susceptibility for T2DM in different races.

Network meta-analysis allow indirect comparison of evidence, which has previously published.^63^ Here, we used the paired-wise method in order to estimate, for each study, the risk of metabolically unhealthy vs healthy individuals in different BMI categories. This method makes comparison of different categories with each other possible which could be considered as one of strength of present study. However some points must be considered before making any solid conclusion about present findings. Although several studies provided longitudinal data on the risk of developing T2DM in different obesity phenotypes, it is difficult to straightly compare results of studies because no consensus has been achieved yet concerning the best criteria for definition of metabolic health.^40,64^ On the other hand definitions used for the diagnosis of diabetes differed among studies.^34,38,65,66^ Different definitions allowed only indirect comparisons phenotypes prevalence in different populations which indicated highly inconsistent prevalence among different studies.^67,68^ Other possible factor which has not been evaluated in present study is that for example, liver fat content mostly explains different risk rates between MHO and MUHO,^69^ in fact presence of non-alcoholic fatty liver disease has led to different risk rate among individuals for T2DM development even in same obesity phenotype.^70^ So in future, other possible confounding factors like the degree of inflammation must be considered.^71^ At last it must be noted that MHO might be a transient setting ^72^; thus, the influence of changes in obese phenotypes on the risk of developing T2DM should be investigated in future studies. The significance to consider for the time-varying nature of these exposures is also reported in meta-analyses, which found elevated risks especially among studies with longer follow-up.^40,73^ Duration and degree of obesity are related to become MUHO^74^ which could be a reason for the diverse results among previous studies.^40,67,73^

## Conclusion

In conclusion we found that being classified as overweight and obese increased the risk of T2DM in comparison with normal weight. In addition metabolically unhealthy individuals are at higher risk of T2DM in all categories of BMI compared with metabolically healthy individuals. MUHO showed higher risk for T2DM development than MHO and MHNW which shows that metabolic abnormalities might contribute to the development of T2DM more than BMI. Furthermore, MUHNW showed increased risk for T2DM development than MHNW which is also higher than MHO and MHOW. Interestingly, the risk for MUHNW compared with MHNW was also higher than MUHO and MUHOW comparing with MHNW, which must be considered in future studies.

## Competing interests

There was no competing financial interest in relation to current study.

## Ethical approval

Not applicable.

## Supplementary files


Supplementary file 1 contains Figure S1 and Tables S1-S5.

## Acknowledgments

This study was supported by the Student`s Scientific Research Center, Tehran, Iran.
