# Supplementary material for: Risk of type 2 diabetes in metabolically healthy people in different categories of body mass index: an updated network meta-analysis of prospective cohort studies
Source: J Cardiovasc Thorac Res. 2019 Oct 24;11(4):254–63. doi: 10.15171/jcvtr.2019.43 (PMC6891044; doi:10.15171/jcvtr.2019.43)
Supplement: Supplementary file 1 — contains Figure S1 and Tables S1-S5. [file jcvtr-11-254-s001.pdf]

Supplementary file 1

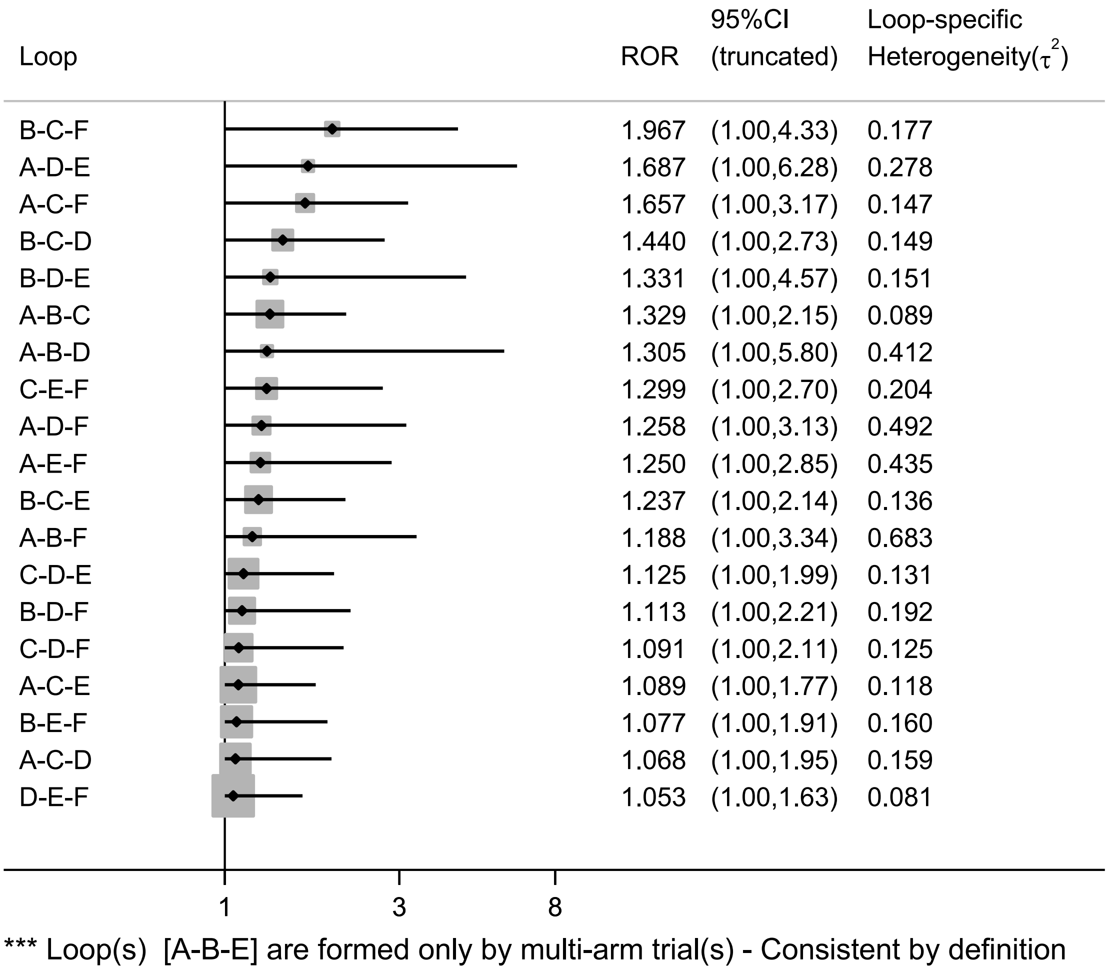

Figure S1. Evaluation of loop specific consistency in effect estimates in triangular and quadratic treatment Loops within each network for the association between BMI and diabetes incidence. A: metabolically healthy normal weight, B: metabolically healthy obesity, C: metabolically healthy overweight, D: metabolically unhealthy normal weight, E: metabolically unhealthy obesity, F: metabolically unhealthy overweight

Table S1. Cohort studies investigation incident type 2 diabetes in metabolic health and body mass index categories									
First Author; YEAR	Country/ ethnicity	Definition Of metabolic health	BMI Categories (kg/m^2)	Study population	Sample size / incident cases	Incident T2D diagnosis	Length of follow-up	Adjustment	Quality score
Meigs 2006	USA/ American	MetS, 3 / 5 ATPIII criteria: FG < 5.6, WC ≤ 88 102 M or 88 W, TG < 1.7, HDL ≥ 1 M or 1.3 W, BP < 130/85	BMI NW<25,OW=25-29.9,OB≥ 30	Offspring of community-based study; European ancestry; free from CVD and T2D at baseline; mean age, 54 years; women,55%	2902/141	FG≥7.0 mmol/l or new use of hypoglycaemic therapy	11y*	age,sex,family history of diabetes,impaired glucose tolerance	8
Arnlov 2011	Sweden / European	MetS: 3 / 5 ATPIII criteria: FG < 6.1c, BMI ≥ 29.4d,TG < 1.7, HDL ≥ 1.04, BP < 130/85	BMI NW<25,OW=25-29.9,OB≥ 30	Community-based study of men free from T2D at baseline; mean age, 50 years, Women,0	(1)1675/160 (2)1385/117	FG≥7.0 mmol/L at follow-up or data from national hospital-discharge registry	20 y	age,smoking,physical activity	8
Hadaegh 2011	Iran/ Asian	MetS: 3 / 5 harmonised criteria: FG < 5.5, WC < 94.5, TG < 1.7, HDL ≥ 1.04	BMI NW<25,OW=25-29.9,OB> 30	Population based cohort study in Tehran; mean age, 42 years; women, 58%	5,250/369	Self-reported or OGTT-based at two follow-up visits	6.5 y	age, family history of CVD,education,smoking,intervention	9

		M or 1.3 W, BP < 130/85							
Kim<br>2012	Korea/ Asian	MetS: 3 / 5 2009 harmonised criteria: FG < 5.6, WC < 90 M or 80 W, TG < 1.7, HDL ≥ 1 M or 1.3 W, BP < 130/85	BMI NW<23,OW23-27.4.OB≥27	Subjects attending baseline and follow-up visits at Health Promotion Centre; mean age,48 years; women,35%	8,748/308	FG≥7.0 mmol/L or HbA1c≥6.5% or treatment	5 y	age,sex,smoking ,alcohol consumption,physical activity	8
Hwang<br>2012	Taiwan / Asian	MetS: 3 / 5 modify AHA criteria: FG < 5.6, WC < 90 M or 80 W, TG < 1.7, HDL ≥ 1 M or 1.3 W, BP < 130/85	BMI NW=18.5-22.9,OW=23-24.9,OB1 =25-26.9,OB2 ≥27	Taiwanese Survey on Prevalences of Hypertension, Hyperglycemia and Hyperlipidemia (TwSHHH); age, 18-59;women, 59.3%	1547/ NR	FG≥7.0 mmol/L or HbA1c≥6.5% or treatment	5.4 y	age,smoking status,alcohol intake status,exercise,family histoy of diabetes and hypertensio	9
[a]BO<br>2012	Italy/ European	MetS plus IR: 3 / 5 harmonised criteria: FG < 5.6, WC < 94 M or 80 W, TG < 1.7, HDL ≥ 1 M or 1.3 W, BP < 130/85 AND HOMA-IR < 2.5	Lean<25, OW 25-30<br>Obes>30	Caucasian volunteers from Local Health Unite; mean age, 54 years; women, 53%	1,658/72	Self-reported, FG, demographic registries	9 y	Age,sex,fiber intake,exercise level,smoking habitest,cardiovascular risk score and waist circumference values	8
Appleton<br>2013	Australia/ Oceania	MetS: 3 / 4 IDF criteria: FG < 5.6, TG < 1.7, HDL ≥ 1 M or 1.3 W, BP < 130/85	BMI NW=18.5-24.9,OW=25-29.9,OB≥30	Community-based study; Adults of European ancestry;	2315/112	Self-reported doctor diagnosis or FG≥7.0 mmmol/L	Median 8.2 y	age,sex,household incom,family history of diabetes	9

Soriguer 2013	Spain/ European	MetS plus IR: 3 / 3 criteria: FG < 6.1, TG < 1.7, HOMA-IR < 90th percentileg	BMI NW<25,OW=25-29.9,OB≥30	Population based cohort study; mean age,40 years; women,62%	378/38	Self-reported or FG at follow-up	11 y	age.sex.weight change , IGT, IFG	8
Heianza,Y 2014	Japan/ Asian	MetS: 3 / 4 IDF criteria: FG < 5.6, TG < 1.7, HDL ≥ 1.03 M or 1.29 W, BP < 130/85	BMI NW<23,OW23-27.4.OB≥27.5	Cohort study of Japanese government employees; ; mean age, 48years; women,27%	8,090/274	FG≥7.0 mmol/L or HbA1c≥6.5% or self-reported	5 y	age , sex parentral history of diabetes, smoking habit ,physical activity ,alcohol consumption,IFG,fatty liver	10
Aung 2014	USA American	MetS plus IR: 4 / 5 ATPIII criteria: FG < 5.6, TG < 1.7, HDL ≥ 1 M or 1.3 W, BP < 130/85, HOMA-IR ≤ 5.13	BMI NW<25,OW=25-29.9,OB≥30	Population based cohort study of Mexican and Caucasian Americans; mean age,42 years; women,57%	2,814/262	OGTT or medication at follow-up	7.4 y	age ,sex,ethnic origin , smoking ,family history of diabetes,fasting glucose	9
Rhee 2014	Korea / Asian	MetS plus IR: 3 / 4 Wildman criteria: FG < 5.6, TG < 1.7, HDL ≥ 1 M or 1.3 W, BP < 130/85 AND HOMA-IR < 90th percentil	BMI NW<23,OW23-27.4.OB≥27.5	Participants of medical check-up programme; mean age, 43years; women,27%	6,748/277	FG≥7.0, HbA1c or self-reported history or medication	4 y	history smoking and education , change of body weight, alcohol drinking ,regular exercise status,antihypetensive medication ,during follow up	8
Twig 2014	Israel/ Asian	MetS: 3 / 4 ATPIII criteria: FG < 5.6, TG < 1.7, HDL ≥ 1, BP <130/85	BMI NW<25,OW=25-29.9,OB≥30	Cohort study of men from the Israel Defence Forces; mean age, 31 years; women,0%	33,939/734	FG or physician diagnosis	6.1 y	age,family history of diabetes,region of origin,physical activity,FPG,TG and WC	8

Hinnouho 2015	UK/ European	MetS: 3 / 4 ATPIII criteria: FG < 5.6, TG < 1.7, HDL ≥ 1.04 M or 1.29 W, BP < 130/8	BMI NW=18.5-24.9,OW=25-29.9,OB≥30	Cohort study of office workers in central London; mean age, 49 years; women, 30%	7,122/798	OGTT or physician diagnosis or use of medication at follow-up	Median 17.5 y	sex,socioeconomic status,marital statuse, ethnicity,physical activity,smoking ,alcohol,fruits , vegetables consumption ,CVD medication	10
Heianza,Y 2014	Japan/ Asian	MetS: 3 / 4 IDF criteria: FG < 5.6, TG < 1.7, HDL ≥ 1.03 M or 1.29 W, BP < 130/85	BMI NW<25,OB≥25	Cohort study of individuals occupational health examinations; mean age, 47years; women,36%	27,891/1,668	FG≥7.0 mmol/L or HbA1c≥6.5% or self-reported	8 y	age,sex,smoking habit ,physical activity	8
Jung 2015	Korea/ Asian	MetS: 2 / 4 ATOIII criteria: FG < 5.6, TG < 1.7, HDL ≥ 1 M or 1.3 W, BP < 130/85	BMI NW<25,OB≥ 25	Cohort study of employees of large Korean company and their spouses; ; mean age, 37 years; women,44%	34,994/889	FG≥7.0 mmol/L or HbA1c≥6.5% or medication	5 y	age , sex,systolib BP, dirinking , exercise habit, family history of diabetes,IFG,ALT,GGT,HoMA -IR	8
Luo 2015	China/ Asian	MetS: 3 / 4 DCG Chinese guideline criteria: WC < 90 M or 85 W, TG < 1.7, HDL ≥ 1.04 BP < 130/85, FBG ≥ 6.1 and/or 2h plasma glucose (2hPG) ≥7.8	BF% NW<25% M,<35%f. OB>25% M,>35%f	community-based prospective cohort study in Shanghai communities—Shanghai Diabetes Study (SHDS); age, 30-90 years; women 57%	2,764/100	Impaired glucose regulation (IGR), including impaired glucose tolerance (IGT) or impaired fasting glucose (IFG)	3.6 y	age,total cholestrol,TG,family history of diabetic disease	8
[b]Kim 2016	Korea/ Asian	MetS: 3 / 4 ATPIII criteria: FG < 5.6, TG < 1.7, HDL ≥ 1 M	BMI NW<25,OB≥25	Population based Cohort study of Korean Genome Epidemiology study;age,40-69; women,	7588/?	OGTT or FG≥7.0 mmol/L or self-reported history or medication	10 y	age , sex, study site, smoking habit ,physical activity ,alcohol intake	9

		or 1.3 W, BP < 130/85,							
Ryoo 2015	Korea/ Asian	Met:3/4 Wildman criteria FG < 5.6, TG < 1.7, HDL ≥ 1, BP < 130/85 AND HOMA-IR C90th percentile.	BMI NW<25,OW=25-29.9,OB≥30	Subjects who visited at Health Promotion Centre for a medical check-up; mean age, 43 years; women 0%	31,834/2,853	FG≥7.0 mmol/L or HbA1c≥6.5% or self-reported history or medication	5 y	age,TG,GGT,eGFR ,smoking status,alcohol intake,regulare exercise ,hypertension	9
Latfi 2017	Iran/ Asian	MetS: 3 / 4 ATPIII criteria: FG < 5.6, TG < 1.7, HDL ≥ 1 M or 1.3 W, BP < 130/85, WC < 102 M or 88 W	BMI NW=19-24.99, OW=25-29.9 OB≥30	Subjects who were selected using cluster sampling in health centers; mean age,42.5 years; women 52.5%	591/ NR	NR	5 y	age,sex,physical activity,BMI, family history disease	8

Abbreviations: y, years; NR, Not reported; M, men; W, women; OW, overweight; BMI, body mass index; T2D, type 2 diabetes; MetS, metabolic syndrome; IR, insulin resistance; ATPIII, Adult Treatment Panel III; FG, fasting glucose; HDL, high-density lipoprotein cholesterol; BP, blood pressure; TG, triglycerides; WC, waist circumference; OGTT, oral glucose tolerance test; WBC, white blood cells; IDF, International Diabetes Federation; 2hPG,2hours plasma glucose; HOMA-IR, Homeostatic model assessment – insulin resistance; BF %, body fat

All cut-off values reported as, mmol/L for fasting glucose, HDL cholesterol or triglycerides, cm for waist circumference, mmHg for blood pressure and kg/m2 for BMI

[a]We did not enter this study in our analyses because did not report the risk of incidence of diabetes and just reported risk of hyperglycemia

[b]This study was not enterd in analyses because reported the incidence of diabetes in phenotypic changes

**Table S2.** Network estimated relative risk (95% CrI) of BMI categories on incidence of diabetes in Asian populations					
MUHOW	_MUHO_	_MUHNW_	_MHOW_	_MHO_	_MHNW_
MUHOW	1.44 (1.02,2.05)	0.65 (0.45,0.93)	0.28 (0.19,0.42)	0.39 (0.27,0.56)	0.15 (0.11,0.21)
0.69 (0.49,0.98)	MUHO	0.45 (0.33,0.61)	0.20 (0.13,0.29)	0.27 (0.20,0.37)	0.10 (0.08,0.14)
1.55 (1.08,2.22)	2.23 (1.65,3.03)	MUHNW	0.44 (0.30,0.65)	0.60 (0.43,0.83)	0.23 (0.17,0.31)
3.52 (2.36,5.25)	5.08 (3.48,7.41)	2.27 (1.54,3.34)	MHOW	1.37 (0.92,2.04)	0.53 (0.36,0.77)
2.57 (1.77,3.73)	3.71 (2.72,5.07)	1.66 (1.20,2.30)	0.73 (0.49,1.09)	MHO	0.39 (0.28,0.53)
6.67 (4.71,9.44)	9.63 (7.22,12.86)	4.31 (3.19,5.83)	1.90 (1.31,2.76)	2.59 (1.90,3.54)	MHNW

**Table S3.** Network estimated relative risk (95% CrI) of BMI categories on incidence of diabetes in American populations					
MUHOW	_MUHO_	_MUHNW_	_MHOW_	_MHO_	_MHNW_
MUHOW	1.70 (1.30,2.23)	0.45 (0.26,0.76)	0.18 (0.13,0.26)	0.38 (0.25,0.57)	0.09 (0.06,0.14)
0.59 (0.45,0.77)	MUHO	0.26 (0.16,0.44)	0.11 (0.08,0.15)	0.22 (0.15,0.33)	0.05 (0.04,0.08)
2.24 (1.31,3.82)	3.82 (2.27,6.41)	MUHNW	0.40 (0.23,0.71)	0.84 (0.46,1.54)	0.20 (0.11,0.37)

5.57 (3.88,8.00)	9.50 (6.78,13.31)	2.49 (1.40,4.41)	MHOW	2.09 (1.32,3.31)	0.51 (0.32,0.81)
2.67 (1.77,4.02)	4.54 (3.07,6.72)	1.19 (0.65,2.18)	0.48 (0.30,0.76)	MHO	0.24 (0.15,0.40)
11.01 (7.25,16.70)	18.76 (12.62,27.89)	4.92 (2.67,9.05)	1.97 (1.24,3.14)	4.13 (2.49,6.84)	MHNW

Table S4. Network estimated relative risk (95% CrI) of BMI categories on incidence of diabetes in European populations

MUHO	_MUHNW_	_MHOW_	_MHO_	_MHNW_
MUHO	0.41 (0.32,0.52)	0.17 (0.12,0.22)	0.29 (0.22,0.37)	0.10 (0.08,0.12)
2.44 (1.91,3.12)	MUHNW	0.40 (0.30,0.54)	0.70 (0.54,0.91)	0.23 (0.18,0.30)
6.04 (4.55,8.02)	2.48 (1.84,3.34)	MHOW	1.73 (1.28,2.34)	0.58 (0.43,0.77)
3.48 (2.72,4.47)	1.43 (1.10,1.86)	0.58 (0.43,0.78)	MHO	0.33 (0.26,0.43)
10.49 (8.31,13.24)	4.31 (3.36,5.52)	1.74 (1.31,2.31)	3.01 (2.34,3.87)	MHNW

MHNW MHO MHOW MUHNW MUHO

Table S5. Comparison between direct and indirect evidence for the association between BMI and diabetes incidence. A: metabolically healthy normal weight, B: metabolically healthy obesity, C: metabolically healthy overweight, D: metabolically unhealthy normal weight, E: metabolically unhealthy obesity, F: metabolically unhealthy overweight

Side	Direct		Indirect		Difference		
	Coef.	Std. Err.	Coef.	Std. Err.	Coef.	Std. Err.	P>z
A C *	0.5855764	0.1601174	0.5025374	0.3644033	0.083039	0.3989535	0.835
A D *	1.471692	0.127286	0.7013871	0.936091	0.7703054	0.9434316	0.414
A F *	2.043931	0.1563395	1.659672	0.359837	0.3842589	0.3955665	0.331
B C *	-0.6363615	0.1734239	-0.0654247	0.3729727	-0.5709368	0.4243033	0.178
B D *	0.3612654	0.1370418	0.32853	0.9317345	0.0327353	0.9429342	0.972

B F *	0.8985897	0.1723678	0.8178452	0.3789386	0.0807445	0.4311686	0.851
C D *	0.8525744	0.171451	1.032575	0.3763911	-0.1800007	0.4241415	0.671
C E *	1.802418	0.1583511	1.634413	0.3592924	0.1680054	0.3921941	0.668
C F	1.327667	0.163449	2.075957	0.4728575	-0.7482902	0.5002455	0.135
D E *	0.9015688	0.1259212	0.0380552	0.9688906	0.8635136	0.9754909	0.376
D F *	0.4751433	0.1685817	0.7293887	0.3764129	-0.2542455	0.4243813	0.549
E F *	-0.398043	0.1546105	-0.198714	0.3594226	-0.199329	0.39277	0.612
